# Local Anæsthesia by Congelation in Dental Surgery

**Published:** 1855-10

**Authors:** J. Richard Quinton

**Affiliations:** London.


AMERICAN JOURNAL

OF

### DENTAL SCIENCE.

Vol. V. NEW SERIES—OCTOBER, 1855. No. 4.

### ORIGINAL COMMUNICATIONS.

### ARTICLE I.

*Local Anæsthesia by Congelation in Dental Surgery.* By J. RICHARD QUINTON, London.

IT is a singular coincidence that there should have appeared in one and the same number of this Journal, sentiments both favorable and adverse to the induction of anæsthesia in dental surgery. In the last April number, Mr. Robinson takes up the cause of anæsthesia, in a leading article, while Dr. Dwinelle, in his valedictory address to the dental graduates, cursorily says, "Let me caution you never to use anæsthetics in your practice, except under circumstances of extreme necessity." We may view these two advocates of an essentially diverse practice, as expositors of certain great principles, and as the media of certain important confessions. Let us abolish pain at any cost, says the one—withhold your hand from that poisoned cup, says the other. An anæsthetic in dental practice is highly desirable, says the one—but the other replies, your anæsthetic is unsafe to your patient's life, and jeopardises your moral reputation; if you must give it, then let it be "only in the presence of a third person; you owe this to your patients, and your profession, as well as to yourselves," (p. 219,) if you do not kill your patient right out, remember, that in that artificial sleep lascivious dreams may come, which, as in more than one instance, may wear the aspect of reality. The question as it now stands between these two expositors, who may be considered as representing the feelings of two great classes of the community, is, the abolition of pain at grave risks, or a continuance in the old method of inflicting pain without alleviation. Whatever may be the real state of the question, such is the form it assumes in the public mind.

Pain is ever repugnant to humanity. It is necessarily so—wisely so. Especially that artificial pain, which, in the exercise of our profession we unavoidably induce by surgical interference. The pain of tooth extraction is by no means the least severe in surgery. It is the object of terror to most. The nervous patient, through the mental dread of what must come, suffers an hundred extractions, all probably affecting and lowering the tone of the nervous system. This expectant suffering, shadows forth the magnitude of the reality. Who is there that would not be spared such pain? Yet how rarely, in this country at least, are the accessible means resorted to, either by dentists or patients, which might spare that pain. A great confession lies at the bottom of this. Has humanity departed from our art? Are the dentist's nerves steeled against a refined sympathy for the suffering? We do not believe it. We believe that our dentists do not find in etherization an unmixed good. Even Mr. Robinson tells us, in effect, that it is very easy by etherization to invade the sacredness of human life; that we may trespass on forbidden grounds: and that, if we do so trespass, we should take with us "the patient's medical adviser;" and have a cabinet of paraphernalia at hand, "sponges and cold water," "diffusible stimulants," "jars of oxygen gas," "galvanic batteries," &c., and all these, notwithstanding the practitioner has served a due preliminary apprenticeship in etherizing "the lower animals," and noting in them that "debility," that "asphyxia," and that melancholy "reaction," which at any moment he may be called upon under similar circumstances to witness in the human subject. And if even an experiened etherizer finds it necessary to have such an array of resources at hand, can we wonder that timorous patients should conclude, that an awful gloom of uncertainty surrounds this artificial unconsciousness. If, when any one of thirty-two teeth turns rebellious, a cabinet council must be held, composed of patient, the patient's doctor, and the patient's dentist, if the pulse is to be noted by the finger, the heart invested by the stethoscope, and the lungs percussed and auscultated, all for the sake of saving what may be but a brief though confessedly severe pain, it must appear to the commonest sense, that a great disparity exists between the means proposed and the end to be obtained. The question must occur, as it has occurred again and again, *is it justifiable to administer chloroform in dentistry?*

The answer to this question depends in part upon the estimate formed of the risks incurred. Individually, I may value these risks as exceedingly trifling in competent hands. Considerable experience in the use of this anæsthetic agent may have led me to see in it far less of the destroyer than the fancies of others seem to elicit from it. But at the same time several considerations present themselves.

"First, the pain of tooth-extraction, though severe, is brief in duration. In numerous instances, the mere pain itself rarely is productive of graver consequences than the momentary shock. In persons highly susceptible of pain, or in certain states of constitution, it is doubtless often of the utmost importance to obviate even this short duration of pain, though perhaps not at a possible sacrifice of life itself. Secondly, it is not ordinarily an operation attended with very grave results. It is not often that the mortality tables are increased by untoward results in dental surgery. The wound caused by extraction is in its nature comparatively slight, and easy of reparation. There is no large mass of tissue to be united, no structures likely to take on extensive inflammation, no pyohæmia, nor phlebitis; no deleterious influence, in fact, bears upon the general system capable of producing the great mortality of many surgical operations. In many of the capital operations, for example, the chances of recovery are extremely small, and indeed in some they are almost reduced to *nil.* The difference then lies in this, that in many surgical operations fatal results are *expected* to occur in certain proportions; while in tooth-extraction such a result may almost be said never to occur. A patient, yielding himself to the doubtful result of returning to life under chloroform in the one case, only throws away so many chances as he might have possessed from the peculiar nature and attendant mortality of the operation itself; while in the other case, the mortality being *nil*, he makes, in inhaling chloroform, an unconditional and unqualified surrender to the possibility of death. The value of a man's life, in the one case, is but as 1 in 2, 3, 4, or 5, and so on; whilst in the other, it is equal to that of the average of the community to which he belongs. Now, putting aside the consideration whether a man has any moral right to trifle with the destinies of human existence, we say, viewing the subject in this double aspect, viz.—the value of any given life under the two kinds of operation, and the brief duration of pain sought to be escaped from, that it is very open to debate if it be justifiable in such an operation as tooth-extraction, to resort to an agent to produce insensibility, which may result in the loss of life. It is this conviction, we have no doubt, which has ever prevented the general adoption of etherization in dentistry. If a man undergoing an operation, knows that his chances of life are small, and that the severity of the accompanying pain is such as to diminish even the few chances he has, then he may, perhaps, in the *absence of better means*, legitimately resort to chloroform; but on the other hand, if his chances of life are equal to those of the general community, and the accompanying pain of the operation is of but brief severity, may he legitimately yield himself to such an anæsthetic agent? The circumstances attending the loss of a thigh may warrant the risk of life by anæsthesia; but to say that the circumstances attending the loss of a tooth should be put on equality with the *possible loss of life*, appears an anomaly, at which our moral sentiment recoils."

Fortunately, we are now relieved from the difficulty of the question, by the introduction into dental practice of a *local* anæsthetic agent, which, while it is efficient, is open to none of the grave objections of etherization; viz. the *anæsthetic application of intense cold.*

For several years past the attention of surgeons in this country, and in France, has been directed to congelation as an available anæsthetic in minor operations; and the great success which has attended its employment, has given it high rank among the humane appliances of surgery; and the more prominently, because its influence is purely local. It admits of no dispute that a great advance is made in anæsthetic science, when the unhappy victims of the surgeon's art, can look with smiles, unknown where pain exists, on the fleshing of the surgeon's knife on their own bodies.

Then comes the question, is congelation applicable in dentistry? *A priori* everything seemed to contra-indicate its applicability. The ordinary methods indeed of producing insensibility by cold as proposed by its originator, Dr. Arnst, were wholly inapplicable in dental operations. It was not possible to keep thrusting into a patient's mouth, a succession of net bags filled with a freezing mixture, nor to fill the mouth, or even touch a tooth, with a metallic ball previously immersed in a cooled fluid. These may have served their purpose well enough upon the external surfaces of the body, but were useless for such a heated cavity as the mouth, with its peculiar anatomical structure and relations.

It was with these difficulties presenting themselves, that Mr. Blundell and myself instituted a series of experiments in order to obtain a method of applying cold as an anæsthetic in dentistry. The first thing required was to supersede the solid form of the freezing mixture by a *fluid medium.* This was easily accomplished; for, as is well known, by the chemical action of various salts upon finely pounded ice or snow, a fluid results of a temperature varying from 20° above to 40° or lower, below zero. (Fahr.) By combining these in sufficient quantity, we were able to keep and apply *an unalterable temperature* to any given surface. The conduction of heat, which takes place when a freezing mixture is applied in the mouth, is so great and rapid as to render the solid form of mixture ineffective from the raising of its own temperature. But as the *fluid* mixture flows over the part, the heat absorbed by it is at once carried away, and the original intensity of cold is renewed. So that by this means we were able to apply to the mouth an *invariable intensity of cold, and that of unlimited duration.*

An apparatus was constructed, consisting of a reservoir for the cooled fluid or freezing mixture, with tubes to conduct the fluid, through a membranous mouthpiece, adjusted over and around the tooth, and stop-cocks to regulate the flow. With this apparatus we commenced to operate, and with such signal success, as to encourage the prosecution of the new method up to its present complete state.

Thus was the first difficulty overcome ; and thus for the first time was a *fluid current* applied to produce local insensibility by congelation for operative surgery.

For many dental operations, such as the extraction of stumps, and even molar teeth under certain conditions, this comparatively imperfect apparatus was perfectly satisfactory and effective. But another difficulty soon presented itself, confirming my previous expectations. It had always occurred to me that the direct application of an intense cold to such sensitive organs as the teeth, would be productive of a pain severe enough to preclude its use in dental operations. It was consequently with a degree of astonishment, almost amounting to incredulity, that we received the assurance from the first patients submitted to this new anæsthetic, that, not only was the extraction itself painless, but that the application of the cold was equally so. On investigating the peculiarities of these cases, this immunity from the pain of the application, appeared susceptible of explanation. The dreaded fact, however, soon stared us in the face, that wherever vitality still remained in the condemned tooth, or in others in its immediate vicinity, the direct application of an intense cold, was attended with such excruciating pain, as to render its employment both cruel and impracticable. Our first successes, so full of promise, so hopefully cheering, thus met with a check which well nigh blighted all hope. But, nothing daunted by failure, a new series of experiments was instituted, many of them on my own teeth, &c., which experiments terminated in the discovery, that, in order to apply an intense cold to a highly sensitive surface without giving pain, the temperature of the cooling fluid or medium must, in the first instance, be equal to the temperature of the part to be cooled. And further, that the temperature must be diminished by a slow gradation until the maximum degree required is arrived at. Working upon this induction, a variety of apparatus was constructed to secure the required graduation of the cooling fluid. Though science and art presented many available modes of accomplishing the object, yet we have found nothing more simple, convenient, and effectual, than plain warm water, that is when the cooling fluid employed in the chemical result of the freezing mixture itself. Thus, for example, suppose the reservoir to contain a due admixture of pounded ice and salt the temperature of which is zero. Let the fluid traverse in its course a vessel of warm water, a mutual diffusion of temperatures takes place, and the result is, that a gradual diminution of temperature will be gained at will, from any degree of the thermometer down to zero. With a similar apparatus to this our first essays were made, and we had the gratification of finding the working of it perfect. Commencing at about blood-heat we proceeded to apply the cooling fluid to the tooth of a patient, (after numerous trials on my own person,) which tooth was full of vitality, and the gratifying result was, not only the *painless extraction* of the tooth, but the *painless application of the cold.* From that time, nearly twelve months ago, to this, a large number of successful operations, has most unequivocally demonstrated, that the time has now come, that we may save our patients the agonies of tooth extraction, without for a single moment depriving them of their valued consciousness.

The work was not, however, completed, though the great object was attained. It would be tedious to give in detail the minutiæ of such endeavors; the difficulties which had to be surmounted, the mechanical contrivances which had to be devised, &c. I will make allusion only to one, because it has been made the subject of remark in an article upon this topic by Mr. Robinson. That gentleman states, that in his attempts to imitate our discoveries, he found his "fowls' stomachs" and "sausage skins" discharge their contents into his patients' stomachs and thereby nauseate them. No part of the apparatus was so difficult of contrivance, as that intended to surround the tooth. *Tenuity* was needed in order to conduct with sufficient readiness the temperature of the cooling fluid to the part to be benumbed; *flexibility* was essential to its perfect adaptation to the varying forms of the teeth and gums; *impermeability* was required to prevent the contained fluid issuing into the mouth or down the esophagus; while a certain elasticity, or resistance of pressure, was necessary, to prevent a rupture of the reservoir, either by the force of the current, or by coming in contact with roughened points or tubercles of the teeth. All membranous structures, like those of animals' stomachs or intestines, are open to the fatal objection, that they become pervious in parts where the blood vessels ramify, as well as that throughout their whole surface they allow of exosmosis. Whenever these were used, the patient always could taste the saline character of the freezing mixture, which should not, and upon my plan, does not occur. Membranes, whether taken from a "fowl's stomach," a "pork sausage," or the "intestine of a rat," while they are flexible, are neither very attenuated nor impermeable, nor elastic, nor, it may be added, of very refined taste. After trying almost every known material, I at length succeeded in making a preparation of india rubber from a solution in chloroform, out of which exceedingly attenuated, almost transparent tubes of any size are made, having all the essential properties above referred to. In consequence, it never happens in my practice to administer to my patients the emetic which Mr. Robinson finds so common in his practice.

Such, in brief outline, is the history of the introduction of anæsthesia by cold in dental surgery. What says experience. I could fill a volume with testimonials, both from professional men and private patients, which have been forwarded me during the past twelve months, under the feelings of spontaniety, aroused by relievance from one of the dreaded tortures of our common lot. I will adduce only a few.

The following letter handed me by a patient, I adduce, because of its conclusive evidence of the value of this method. The writer of it had two large molar stumps extracted, one under the influence of congelation, the other *without* any benumbing application; with what results, let the letter testify.

"I cannot withhold giving expression to my experience of the great boon conferred on society by your excellent method of extracting teeth without pain. Having been a great sufferer for a long period from two firm and large stumps, the extraction of which I had for many months deferred from the dread of the operation, I hailed the announcement of your method with much joy, and at once resolved to place my case in your hands. I did so; not, however, without some secret misgivings; for I was well aware that my teeth would have to be punched out, and I confess to a participation in the general horror of that necessary method of operating. I am only too glad to be able to testify, that under your new method, that horror is done away with. My two stumps, you will remember, had to be taken out separately. To the first you applied your method of congelation, and *with such success that I only knew it had been extracted from the fact of seeing it out of my mouth.* I had fully expected that the application of the cold itself would have been as painful as the extraction; but the congelation was evidently so gradually effected, that beyond a trivial sensation of cold I felt nothing. A sadder experience, however, awaited me. An accident happening to your apparatus, you prevailed upon me to submit to the extraction of the remaining stump without its being previously put under the influence of congelation, and the pain attendant upon that operation (though apparently less difficult to extract than the other) I shall not soon forget. At one and the same sitting, I thus had the double experience of what tooth extraction is under your process, and what it is in the ordinary way. Under such peculiar circumstances, I, at least, can have no doubt, nor can I be under any delusion, respecting the efficacy of your method. I have abundant reason to extol this new and excellent discovery. It cannot be too widely made known for the benefit of mankind; more especially as there was no alternative for me than to endure the pain or take chloroform; but my dread of chloroform nothing could overcome. I dare not inhale it under any circumstances, much less to destroy the brief pain, torturing though it be, of having a tooth extracted; I could not risk my life upon such an unequal balance. And I am satisfied that I am but one of a large class (of timidities, perhaps you will call us) who entertain the same fears of any agent which, to destroy pain, must destroy consciousness, and who, I am sure, will hail this discovery with delight."

In the following case, the patient on *three* separate occasions submitted to the production of local insensibility by congelation, losing in all *ten teeth* without pain.

"I cannot resist thanking you for relief from, to my mind, one of the horrors of life, so almost universally experienced, viz. from the pain of tooth extraction. No one can, I think, have put your valuable process to the test more than I have done, and no one can be more delighted with the results, which have been satisfactory beyond my highest expectations. When I first visited you (which I confess to have done with little hope of such good results,) there were removed four stumps of considerable size and firmness, and one large back tooth, and I can truly say that, *the only sensation I felt, was like that of drawing a finger from a well fitting glove.* Encouraged and rendered fearless by this success, I placed myself a second time in your hands, on which occasion a large stump and another large tooth were extracted with equal freedom from pain. On a third visit, three other large stumps were taken out, and without any suffering whatever. Altogether, I have had ten teeth extracted under your process, and with such abolition of pain as to do away with that dreadful nervousness so natural when compelled to submit to such operations. Your method of applying the cold is to me as surprising as its beneficent effect of annulling pain; for the entire process was to me wholly free from suffering, though I believe you informed me when applying it, that the degree of cold applied was as low as zero, yet I could not perceive it. But little bleeding followed, and the open spaces healed up beautifully. Success must attend such a valuable method of destroying pain. Its benefits only require to be known, to be valued by all who like myself turn shy at pain."

The ensuing letter testifies to two very important features, which I have repeatedly witnessed in my practice, viz. first, that the anæsthetic application of cold is highly serviceable in dispersing *tooth-ache.* I frequently apply it for this alone, and with considerable success. An aching tooth is set at rest, as I can personally testify, for months, and it may be for years. Secondly, it is the invariable experience of my patients that they are relieved from those feelings of exhaustion and nervous disturbance, which so ordinarily follow tooth extraction.

"Suffering greatly from tooth-ache, it was with a mingling of hope and fear that I resolved upon trying your method of painless extraction, which I had heard so much extolled. My enemy was a bicuspid tooth. Your method of freezing was applied for the space of two or three minutes, and so effectual was the insensibility produced, that *I was totally unconscious that my tooth had been withdrawn until you showed it to me.* I could not have credited that such perfect unconsciousness of the detachment of a tooth was possible, except under the sleep of chloroform. One effect of the cold was peculiar, and worthy, I think, of remark. I entered your consultation room with my tooth aching dreadfully; but, as the power of the cold applied was increased, my tooth-ache gradually diminished till it wholly disappeared. This soothing effect, apart from every thing else, was delightful. But the grand thing of all is, that *the horrid wrench of tooth-drawing is by your method perfectly abrogated.* Being anxious that you should fill up with artificial teeth those spaces in my mouth, (which were so many evidences of what I had previously suffered in the hands of the dentist,) it was found necessary to remove a large lower molar. This was done in the same manner and with the same happy results. Nor should I have hesitated to have had any number of teeth out that may have been requisite, under such a pain-annihilating process. I

am satisfied that in the exercise of such a valuable and beneficent work, you will obtain the enviable position of a dentist unfeared by his patients. Another advantage of your method, which was so grateful to me, was the entire freedom from that nervous exhaustion which had been a source of such misery and suffering to me after all my other extractions. I do not attempt to explain it—it may be from the absence of the pain, and of the ordinary nervous shock; but I know that I left your house with a degree of comfort which I never before experienced when having my teeth taken out. Usually I have hastened home and sought repose; but after having visited you, I was able to pursue, without discomfort, my ordinary avocation."

The following letter I bring forward because it proceeds from the pen of a member of the medical profession, who, like many others, was very skeptical upon the subject.

"I place the following account of my tooth and its extraction at your disposal, and should the perusal of this letter inspire with confidence any one disposed to doubt the efficacy of cold, as an anæsthetic in tooth-drawing, the very earnest wish and object of its writer will be accomplished. I purposely abstain *in toto* from anything like persuasive argument, however, on the subject; I merely wish to place before you and all others whom it may concern, the plain and brief facts of my case, which are as follows:

"Two years ago, being tormented with violent tooth-ache in the first molar of the left side, which was in a very carious state, I applied to a surgeon for relief. He applied the key in the usual way to the tooth, but so firmly was it fixed in the alveolus, that it could not be moved with any force that could be safely used. This failing, the forceps were tried, and during this attempt the tooth broke, leaving the two fangs still firmly fixed; the operator was utterly unable to move them, and so they were left in the jaw. These fangs gave no pain till a few months back, when by their irritation they caused frequent abscesses in the gum, attended often with a degree of pain, and always with awkward swelling. In consequence of this I applied to you for relief, and you promised me relief—and painless relief, too. On this latter point, I confess, I was quite skeptical; but with how little reason will appear in the sequel. The stumps were examined, and it was very evident that they were very tight—in fact, a very difficult case of stump extraction; and I myself knowing what tooth extraction was, from previous experience, could not but anticipate some pretty severe pain. Cold was applied gradually, until the gum was rendered bloodless and benumbed, *and this process caused not the least pain.* As soon as this state of gum was thus attained, the forceps were introduced, and one stump grasped and extracted with most admirable skill. I felt no pain worth thinking of; *and certainly could not have believed the stump to be out, until I saw it in the teeth of the forceps.* When examined, it was found curved somewhat at its root, and dilated so as to get a very firm hold of the alveolus; this rendered its extraction longer than it would otherwise have been; and I cannot but think, for my part, that, had a very rapid extraction been attempted, the stump would most infallibly have broken short. The necessarily protracted nature of this extraction sufficiently accounts for the very slight pain felt towards the close. When bleeding had stopped, the cold was applied to the second stump, and though this was broken right down into the gum, and all but, if not quite, out of the operator's sight, yet still the forceps seized it at once, and without a single slip brought it clean out, *without any pain whatever.* The cavity left, I may say, *healed up with the greatest rapidity, in a perfectly healthy way.*

"Such then, sir, are the facts of the case, and I feel much pleasure in writing them out; indeed, I do feel it quite a duty to make them as public as I can, for the sake of individuals who have to undergo the notoriously painful operation of tooth drawing."

To these I may add my own personal experience. Being desirous of arriving at a proper and true estimate of the actual amount of anæsthetic effect produced by congelation upon the teeth, I submitted, for the acquisition of such knowledge, to the loss of a first upper molar. The operation was unusually long and severe. My assistant (the operator) was compelled to use immense force to detach the tooth, owing to the curving inwards of the extremity of the fangs. A more severe case can rarely happen in practice. Yet, I can most confidently assert *that I felt not the slightest pain* throughout this long proceeding.

The experiment took place in the presence of two witnesses, one of them a surgeon dentist; and, having expressed my own experience, I append their evidence of what they saw.

"Most readily we give our testimony to one of the great phenomena of this age. Great was our confidence in your previous assurances of what you had accomplished on others, it has been perfectly eclipsed by the *unmistakeable painlessness* of the dreadful operation we have this day witnessed on your own person. Rarely, indeed, does it fall to the lot of the dentist to perform an operation of greater severity than that to which you were subjected. We feel no hesitation in saying, that under ordinary circumstances it could scarcely have been borne, however great the power of endurance: yet the closest inspection of your countenance could not detect the slightest wince, (and it would be difficult for a dentist to be deceived if pain were present,) you maintained the most perfect calmness and composure throughout the whole proceeding, and we feel bound to affirm that such composure could only be sustained under a perfect *immunity from pain.* The fact which we have witnessed is a proof beyond all doubt that dentistry has come to the humane and painless.

JOHN WHITEMAN WEBB,
Surgeon Dentist.
21 Southampton street, Bloomsbury.

W. H. THORNTHWAITE,
123 Newgate street."

I am indebted to my friend Mr. Webb, of Bloomsbury, for the following additional evidence of the efficacy of this new method in his own practice.

"I have now used your apparatus for producing anæsthesia by cold, in dental surgery, for some time past, and am happy in being enabled to say, I have applied it with great success.

"It appears to me that the great merit of the apparatus consists in its being the most scientific and effectual method of inducing local anæsthesia by congelation; while at the same time insensibility of the part is produced without causing pain to the patient, in fact, it renders the application of the cold as painless as the extraction of the teeth. It is a great fact that we are now able to abolish the pain of tooth-extraction, so much dreaded by patients; and that persons of the most delicate and nervous temperament can bear the operation without any shock to the system, and with freedom from all the stupefying effects produced by chloroform. J. WHITEMAN WEBB."

And lastly, Mr. Robinson affirms in his article on anæsthesia in dental surgery, which appeared in the last number of this Journal, that in nine cases he "*had succeeded in the removal of the teeth without pain*," by the application of cold, although the apparatus he employed on these occasions was signally defective in the one most important particular, which had escaped his notice in imitating that which we have for many months used. The evidence, however, thus given by Mr. Robinson, is worth this much, that tooth-extraction may be rendered painless by the anæsthetic application of cold. And his evidence, taken in conjunction with that emanating from professional men, patients, and other dentists, removes the subject out of the sphere of mere hypothesis or skepticism, into the domain of true science and actual fact.

The *modus operandi* whereby the anæsthetic effect of cold is produced, mainly consists, we believe, in *an arrest of the circulation* in the part to be operated upon, and just in proportion as this is effected, is the amount of diminution of the pain. As the cold fluid continues its course over the gums, the blood-vessels contract, and stop the supply of the circulating fluid to the part, which part consequently assumes a perfect bloodless and blanched appearance. When this state supervenes, all sensibility in the gums is destroyed, so that operating instruments may be used with freedom in this region without the patient suffering any pain. But this is not all that is required. The soft tissue of the gums, succumbs much more readily to the action of the cold, than does the hard bony structure of the teeth or alveolus. If, therefore, the application be stopped when the gums first assume the blanched appearance, and the extraction be proceeded with, there will, in all probability, be more or less of pain attending it. Every substance has its own peculiar power and rate of conduction of heat or cold. It is well known that bone is a slow conductor. Its own time must consequently be given it to conduct the cold, in the case of a tooth, throughout its entire surface from the crown to the apex of the fang. It must not be forgotten that the tooth rests in a bed, wrapped round with membranes through whose vascular tissue, an ever renewing warm fluid is circulating. The power of that recurrent fluid must be overcome; it must be driven back from its accustomed channels, and then, when these membranes are pale and cold, the tooth may be withdrawn without the endurance of that horrid wrench which forms so large a proportion of the dreaded pain. And lastly, the blood being forced out of the vessel entering the apex of the fang by the advancing cold, deprives its associated nerve of its sensitiveness to interference and suffering.

The correctness of this explanation is corroborated by the fact, which experience has amply revealed, that in proportion as the entire tooth is pale and cold when extracted, is the degree of immunity from suffering. In the first case cited above, for example, in which one stump was extracted under the influence of the cold, and another without any such application, the difference, both in temperature and appearance, was very remarkable. The one was pale, bloodless and cold, while the other, which gave so much pain, was highly injected and warm. I adduce this in illustration, as a type of every successful case. The limits of this article do not permit me to enter into detail upon this subject. I hope to be able to lay before the profession, the results of my experiments and experience on the power of conduction, possessed by such structures as those of the teeth, in another paper, which may serve as some guide in the practical application of this new anæsthetic.

But, it may be asked, admitting that by the application of intense cold, we may mitigate or abolish the pain of toothextraction, may it not be productive of serious mischief to the tissues of the mouth? Will not the parts lose their vitality and slough away? No objection to this mode of anæsthesia is more common and none more groundless. In order to determine the force of this ever ready objection, and to ascertain what the actual results of congelation were, I have instituted a series of experiments on the lower animals, and on various surfaces of my own body. I can only here state the general result. On no occasion, though in many instances the congelation was very protracted, have I witnessed or suffered from any symptom more aggravated than an effusion of serum beneath the cuticle. A certain degree of redness has been present, though persistent only for a short time; but no suppuration or sloughing or other symptoms and terminations of inflammation. It were as rational to give the name of inflammation to the suffusing blush of modesty, as to denote by that term the effects of congelation. Dr. Arnstt's experience coincides with my own. "Not an approach," he says, "to inflammation has ever followed the longest continued congelation." I have now employed a degree of cold varying from 10° above zero to 15°, (and often as low as 40°, where inflammation has been present) below zero, in a very large number of dental operations, without meeting with any of those grave results and serious disorganizations, to which fear, ignorance and prejudice had giving imaginary existence. Rarely I have found the epithelial or epidermic covering of the gum peel off, but this has never been a source of inconvenience or injury; being limited only to the immediate vicinity of the part operated upon. The antiphlogistic property of cold itself, is a sufficient answer to all hypothetical conclusions as to its results.

Complications, of course, may arise under the anæsthetic use of cold in dentistry, as in the ordinary mode of practice. These complications may be unfairly put to the account of congelation, as, when chloroform was first introduced, every complication was alleged to be due to the chloroform. To this class belong the two cases of hemorrhage adduced by Mr. Robinson. It was certainly a singular coincidence that two out of nine of his cases should have so resulted. If chloroform had been given in those cases, would Mr. Robinson have attributed the hemorrhage to the chloroform? Hemorrhage, and severe hemorrhage too, is not an unknown occurrence in dentistry. I do not presume to account for the alleged hemorrhage in these cases. I have not met with a single case of the kind in all my practice with congelation. I know not with what show of reason such an occurrence can be at all due to a *properly directed* employment of congelation. In the seven remaining cases recorded by Mr. Robinson, no unusual hemorrhage occurred. And even though some suspicion were admitted to rest upon the congelation as the cause, it must be remembered in the first place, that the operations were performed upon an erroneous principle of applying the cold—one, which might possibly so act upon the blood-vessels as to bring about such a result: and in the second place, that proper care was not exercised in graduating the return of the blood-vessels to their normal condition and functions. The business of the anæsthetiser is not over when he has frozen down the tooth, and extracted it without pain; for, as he has induced a suppression of the normal action of the part, so must he with a wise precision and due attention, *restore* that part to its normal functions.

I wish to claim for congelation as an anæsthetic in dentistry, no more than its true value. That under proper management it is effective in the great majority of the cases which falls into the dentist's hands, experience satisfies me. That it will wholly abolish the pain of extraction in every case I do not at present pretend to affirm, though future improvements may realize even that desideratum. That it must mitigate pain to a considerable extent, in every instance, I feel justified in asserting. Cases ever and anon occur, which I need not enumerate, the severity and duration of which, call for an anæsthetic influence which is more enduring than congelation. In such cases, if desirable to avoid the effects of severe pain, chloroform is preferable; and I do not hesitate to resort to it. But, it will be admitted, that an efficient local anæsthetic must maintain a vast pre-eminence over the abolition of human consciousness.

If the sufferings accessory to dental practice can be abolished, or even mitigated to an easily endurable extent, without any of the inconveniences and risks of etherization, against which the outcry is so loud, and at which dentists in general are so strangely appalled, the humaner tendencies of our art will be amply satisfied. The substitution, in all available cases, of a perfectly harmless congelation, for an anæsthetic which few can inhale without some dread, that in its vapors death may advance with stealthy step, not only meets an almost universal want, but is also in harmony with prudence, reason, justice and humanity.

The following conclusions are fairly deducible from what I have herein advanced:

1. That congelation, by the process I adopt, is efficient for the painless extraction of teeth; or, if not in all cases effecting absolute painlessness, that it so far mitigates the suffering as to deprive the operation of its ordinary repulsiveness.

II. That the peculiar process of congelation, by which this immunity from suffering is accomplished, is itself unattended with pain or inconvenience.

III. That no untoward results accrue from this process of congelation, either local or general. On the contrary, that patients generally enjoy an immunity from the exhaustion usually succeeding tooth-extraction.

IV. That this method is pre-eminently applicable to cases of stumps, the extraction of which is ordinarily so difficult and painful.

V. That the benumbing effect of congelation is often useful in dissipating tooth-ache.

VI. That congelation is infinitely superior to etherization in dental practice, in effecting the same humane purpose with immunity from the natural dread and inconvenience of the loss of consciousness, immunity from alleged possible constitutional effects of chloroform, and immunity from the possible loss of life.

*London, 29 New Broad street, City.*

NOTE.—It is exceedingly painful to me to feel bound to condescend to personal reflections. But as an apparent claim has been made by Mr. Robinson in the pages of a late number of this Journal, to priority in the use of congelation in dentistry, a few comments may not be considered unreasonable or unjustifiable in the same pages. In the preceding article I have given the history of the introduction of congelation as an anæsthetic into dental practice. How comes it to pass, that Mr. Robinson makes no reference whatever to this history, with which he was well acquainted, both by personal curiosity and by printed records *nearly a year old ?* His remarks would lead the readers of this Journal to believe that *he* was the originator of successful anæsthesia by cold in dentistry; and that *he* had contrived original apparatus for that purpose. How far Mr. Robinson can sustain his ground, I gladly leave for the moral sense of the profession to determine. Mr. Robinson adds, "*for general application in dental surgery the new anæsthesia is not at present complete.*" He had applied congelation upon such erroneous principles, that, to use his own words, "*the intense pain produced was indescribable.*" As I have detailed, we ourselves at first fell into the same error, but we speedily remedied it, and in doing so, made our anæsthesia *complete.* It was so *completed* last year. Mr. Robinson knew this. How is it he has not advanced his parallel? Mr. Robinson, with a *quasi* liberalism, wrote, he tells us, "with a view of enlisting the co-operation of the members of the dental art in America." In doing so he made an appeal to professional skill, high intelligence, and a world-esteemed humanity. But the appeal was so far unnecessary, that the work had long before been completed. Nor does it argue much for the strength of Mr. Robinson's *professional fraternity,* that he feels compelled to seek three thousand miles away from home, that aid which was ready at his own door. The fact is, and it cannot be disguised, Mr. Robinson wrote the article in question with the full knowledge that he was asking aid in a matter where no aid was required. Before a word of that article was penned, the new mode of anæsthesia was in full and perfect operation. Mr. Robinson's partner called at our rooms, spied our apparatus, ascertained the mode of action as he conceived; and the result was, the trials alluded to by Mr. R. at the Royal Free Hospital, &c. Unfortunately for Mr. R., and for his patients who were the subjects of these trials, the most important feature of the apparatus was overlooked; hence, though the outward form was copied, those inward workings remained unknown, which would have saved Mr. R. his mistake and his patients their torments. I can readily understand that no man in the dental profession would be more eager than Mr. Robinson to embrace an anæsthetic which obviated the abolition of consciousness, seeing that in his practice, he has I believe, had an unfortunately fatal case of etherization, but this cannot justify any attempt at the appropriation of other men's inventions, without acknowledgment.

Mr. Robinson seems annoyed that any professional man should legally secure to himself the honor which is due to him. I do not wish to enter into the question of the propriety of professional men obtaining patent rights for their discoveries. I would only remark, that, the opprobrium, if it be one, rests upon those pirates who are ever ready to prey upon other people's ingenuity, who, themselves deficient in the qualifications of discoverers or inventors, rob the worthy of the honor which is their due. It rests not upon the man who secures himself against their mean depredations. If there were no such pirates, if honor were given where honor is due, if every man had accorded to him by his brother man, the true reward of his thoughts, his inventions, and his toils, society would want no patent laws, at present they are evidently requisite as police to keep down animals of prey.

